# PW03-004 - PFAPA patient’s serum sensitizes monocytes to LPS

**DOI:** 10.1186/1546-0096-11-S1-A230

**Published:** 2013-11-08

**Authors:** G Simon, N Busso, A von Scheven-Gete, A So, M Hofer

**Affiliations:** 1 CHUV / Rhumatology, Lausanne, Switzerland; 2 CHUV / Pediatrics, Lausanne, Switzerland

## Introduction

PFAPA is a pediatric auto-inflammatory syndrome of unknown etiology, characterized by recurrent fever, aphthosis, pharyngitis and cervical adenitis. Dysregulated monocyte interleukin-1 beta (IL-1β) secretion is thought to play an important role in fever flares.

## Objectives

We hypothesized that factor(s) present in the serum of PFAPA patients during a fever flare may induce monocytes to secrete IL-1β which prolongs symptoms.

## Methods

Serum of three controls (CTRL) or PFAPA patients collected during (PFAPA-IN) and between (PFAPA-OUT) flares were incubated with monocytes isolated from healthy volunteers (n=3) and stimulated with ultra pure lipopolysaccharide (LPSup). IL-1β, TNF-α and IL-6 levels were measured by ELISA comparing serum stimulation alone and the impact of serum pre-incubation on LPSup induced cytokines.

## Results

Serum-alone treatment of monocytes did not induce any detectable increase in IL-1β, TNF-α or IL-6 secretion. However, pre-treatment with PFAPA-IN serum did result in significantly more IL-1β secretion following LPSup stimulation as compared to PFAPA-OUT serum pretreatment (330.8 ± 181.5 and 173.5 ± 100.1 pg/ml respectively; n=9; p<0.05). There was no modulation in the levels of TNF-α and IL-6 under the present conditions, arguing for a process specific for the IL-1β pathway.

## Conclusion

Incubation of healthy donor monocytes with serum collected during a PFAPA flare increases the capacity of monocytes to secrete IL-1β through TLR4 ligation. The identity of the molecule(s) and the mechanism of action of this observation still remain to be elucidated.

## Disclosure of interest

None declared

